# Zika infection of neural progenitor cells perturbs transcription in neurodevelopmental pathways

**DOI:** 10.1371/journal.pone.0175744

**Published:** 2017-04-27

**Authors:** Lynn Yi, Harold Pimentel, Lior Pachter

**Affiliations:** 1 Division of Biology and Biological Engineering, California Institute of Technology, Pasadena, CA, United States of America; 2 David Geffen School of Medicine, University of California Los Angeles, Los Angeles, CA, United States of America; 3 Department of Genetics, Stanford University, Palo Alto, CA, United States of America; 4 Computing and Mathematical Sciences, California Institute of Technology, Pasadena, CA, United States of America; University of Texas Health Science Center at Houston, UNITED STATES

## Abstract

**Background:**

A recent study of the gene expression patterns of Zika virus (ZIKV) infected human neural progenitor cells (hNPCs) revealed transcriptional dysregulation and identified cell cycle-related pathways that are affected by infection. However deeper exploration of the information present in the RNA-Seq data can be used to further elucidate the manner in which Zika infection of hNPCs affects the transcriptome, refining pathway predictions and revealing isoform-specific dynamics.

**Methodology/Principal findings:**

We analyzed data published by Tang *et al*. using state-of-the-art tools for transcriptome analysis. By accounting for the experimental design and estimation of technical and inferential variance we were able to pinpoint Zika infection affected pathways that highlight Zika’s neural tropism. The examination of differential genes reveals cases of isoform divergence.

**Conclusions:**

Transcriptome analysis of Zika infected hNPCs has the potential to identify the molecular signatures of Zika infected neural cells. These signatures may be useful for diagnostics and for the resolution of infection pathways that can be used to harvest specific targets for further study.

## Introduction

As infection with Zika virus (ZIKV) is associated with increasing cases of congenital microcephaly and adult Guillain-Barre Syndrome, a characterization of its pathophysiology becomes crucial. A characterization of the molecular effects of infection may help in the development of fetal diagnostics and can accelerate the identification of genes and pathways critical in disease progression. RNA-Sequencing (RNA-Seq) is an effective technology for probing the transcriptome and has been applied to study the effects of ZIKV infection of human neuroprogenitor cells (hNPCs) [1].

While initial analyses of the data conducted a general survey of transcriptome changes upon infection [1–3], they [1,2] used a method, Cufflinks/Cuffdiff [4], that failed to take advantage of the experimental design used in Tang et. al [1]. They [1–3] also did not examine transcriptome dynamics at the isoform level.

We applied the recently-developed kallisto [5] and sleuth [6] programs to improve the accuracy of quantification and to extract information from the data that was previously inaccessible. We found that sleuth’s improved control of false discovery rate [6] resulted in differential transcript and gene lists that are much more specific and that are significantly enriched in neurodevelopmental pathways. They reveal ZIKV’s neural tropism and the host’s response to viral infection. Furthermore, we demonstrate that the combination of accurate kallisto quantification, assessment of inferential variance and the sleuth response error model allows for the detection of post infection isoform-specific changes that were missed in previous analyses.

The sleuth Shiny app drives a freely available website that allows for reproducibility of our analyses, and provides tools for interacting with the data. This makes the dataset useful for analysis by infectious disease experts who may not have bioinformatics expertise.

## Methods

We ran kallisto and sleuth on a total of eight RNA-seq samples of ZIKV-infected and mock-infected hNPCs (GEO: Series GSE78711) (See Table 1 for experimental design and description of samples). We used kallisto to pseudoalign the RNA-seq reads and perform bootstraps, using an index based on the ENSEMBL GRC38 *Homo sapiens* release 85 transcriptome. For single-end read quantification, we used default parameters (kmer size = 31, fragment length = 187 and sd = 70). For each of the eight samples, kallisto quantified transcript abundances and performed 100 bootstraps.

**Table 1:** Experimental design. Tang et al. infected two samples with ZIKV and two with a mock infection. Library preparation was performed for each sample to make four cDNA libraries. Each library was then sequenced with MiSeq using paired-end reads and NextSeq using single-end reads.

Sample	Accession Number	Condition	Seq method	Seq machine	Reads	No. Fragments / weights
Mock1-1	SRR3191542	mock	paired-end	MiSeq	15855554	7927777
Mock2-1	SRR3191543	mock	paired-end	MiSeq	14782152	7391076
ZIKV1-1	SRR3191544	zika	paired-end	MiSeq	14723054	7361527
ZIKV2-1	SRR3191545	zika	paired-end	MiSeq	15242694	7621347
Mock1-2	SRR3194428	mock	single-end	NextSeq	72983243	72983243
Mock2-2	SRR3194429	mock	single-end	NextSeq	94729809	94729809
ZIKV1-2	SRR3194430	zika	single-end	NextSeq	71055823	71055823
ZIKV-2-2	SRR3194431	zika	single-end	NextSeq	66528035	66528035

The response error model of sleuth was then used to identify differentially expressed transcripts. Sleuth used the bootstraps performed by kallisto to estimate the inferential variance of each transcript, and an adjusted variance was used to determine differential expression for that transcript. This data set had a unique experimental design, however. For each sequencing library corresponding to a biological sample, Tang et al. performed both paired-end and single-end sequencing. To take advantage of the technical replicates performed by Tang et al., we modified sleuth to perform a weighted average of the inferential variance with the number of fragments sequenced (Table 1).

**Fig 1:**
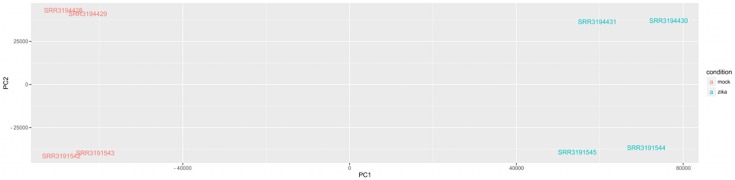
Principle component analysis. PCA of the eight samples shows that the primary contributor to variance is ZIKV infection status (ZIKV vs mock), while the secondary component is sequencing method (paired-end vs single-end).

Principle component analysis of the transcript abundances provided a quick verification of the accuracy of our methods, as the first principle component separated the samples by infection status and the second principle component separated the samples by sequencing method (Fig 1).

The data analysis pipeline was performed on a laptop and can be repeated using the provided scripts at http://www.github.com/pachterlab/zika/. The kallisto quantifications, the modified version of sleuth, as well as a script for the pipeline, are available on the github. One can use the script to start the Shiny app, which recreates the statistics and figures referenced throughout this paper, along with interactive data visualization tools. Alternatively, the preloaded sleuth Shiny app can be found via http://128.32.142.223/tang16/.

## Results

**Fig 2:**
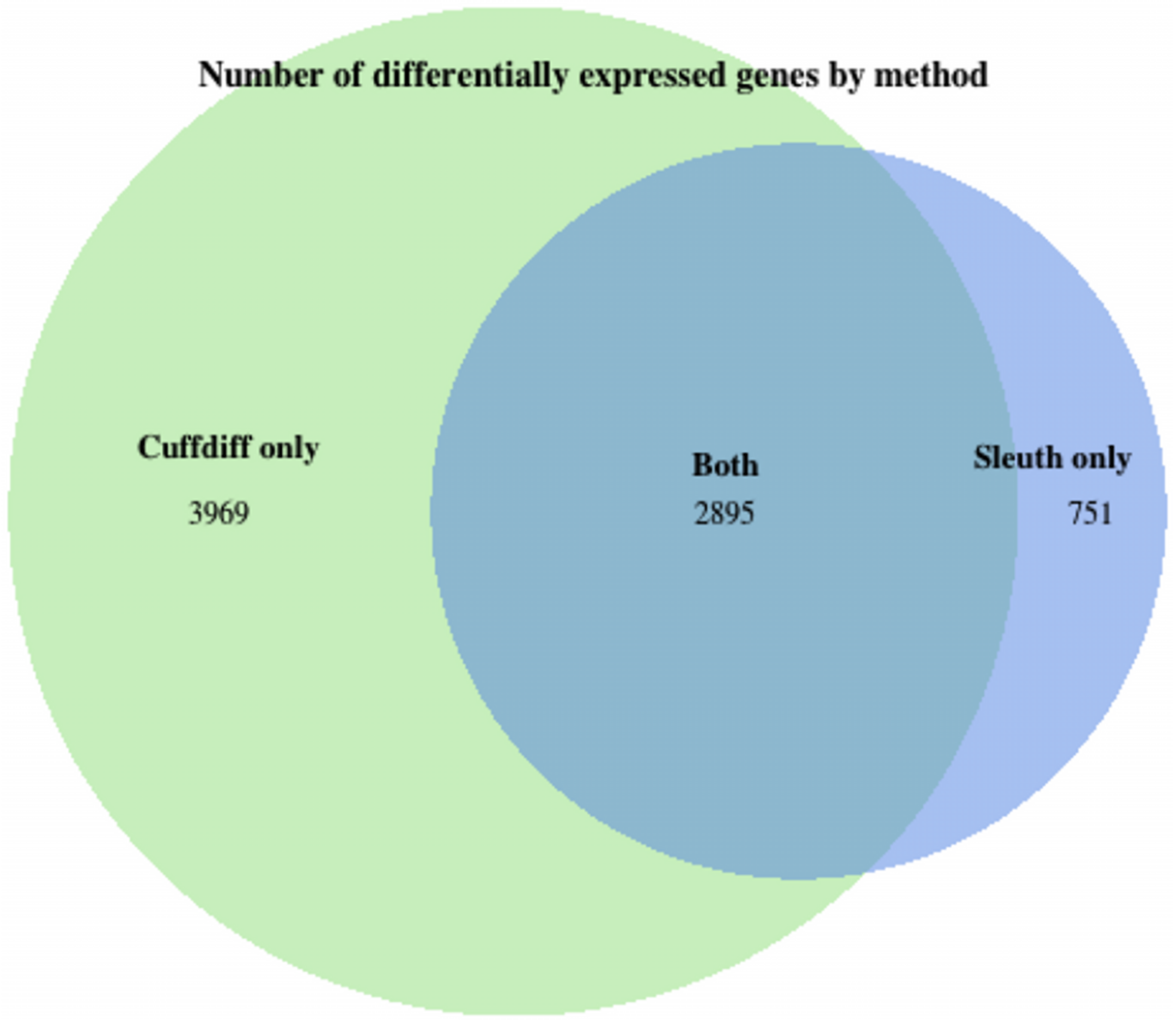
Venn diagram of differential expression analysis. Sleuth identified 3646 differentially expressed genes. Cuffdiff identified 6864 differentially expressed genes. 2895 of the 3646 differentially expressed genes were also reported in Tang et. al [1], but they reported an additional 3969 genes that we failed to identify. Furthermore, we found 751 differentially expressed genes corresponding to 5426 transcripts not detected by Cuffdiff.

Using a false discovery rate of 0.05 as the threshold for differential expression, we detected 4610 transcripts across 3646 genes that are differentially expressed between ZIKV-and mock-infected samples. (Fig 2, S1 and S2 Tables) For the 3969 genes that Cuffdiff found differentially expressed but sleuth did not, sleuth reported an average false discovery rate of 0.55.

It was not surprising that the many differentially expressed genes discovered by Cuffdiff were considered false positives by sleuth. In simulations by Pimentel et al [6], sleuth provided the most accurate false discovery rates, whereas other methods including DESeq2, edgeR, and Cuffdiff2 underestimated their false discovery rates. In other words, these methods provided differential gene lists that had many more false positives than what was suggested by their p-values. The fundamental idea underlying sleuth is that, by using bootstraps to estimate inferential variance, it does not assume a parametric distribution to account for uncertainty in isoform mapping.

Furthermore, we found a few hundred genes with differentially expressed transcripts not identified by Cuffdiff. We ascribe these to the accounting of experimental design and the isoform-level analysis.

### Zika induced isoform divergence

Differentially regulated genes may be missed in gene-level analysis for several reasons. Noise in the measurement of highly expressed transcripts can mask expression changes in lowly expressed transcripts. In the case of isoform switching, upregulation in one isoform and downregulation in another may “cancel out.” We identified 108 genes that contain transcript(s) that are significantly upregulated and other transcript(s) that are significantly downregulated, a phenomenon we coin “isoform divergence” (S3 Table). Of these 108 isoform diverging genes, 57 were not considered differentially expressed by Cuffdiff analysis.

We performed a pathway analysis on the 108 genes using Reactome [7]. Enrichment was identified in neuronal system (specifically transmission across chemical synapses and protein-protein interactions at the synapses), developmental biology (specifically axon guidance), immune system, DNA repair, chromatin modifying enzymes, gene expression (rRNA and transcriptional regulation), metabolism, signal transduction, transmembrane transport and vesicle-mediated transport.

**Fig 3:**
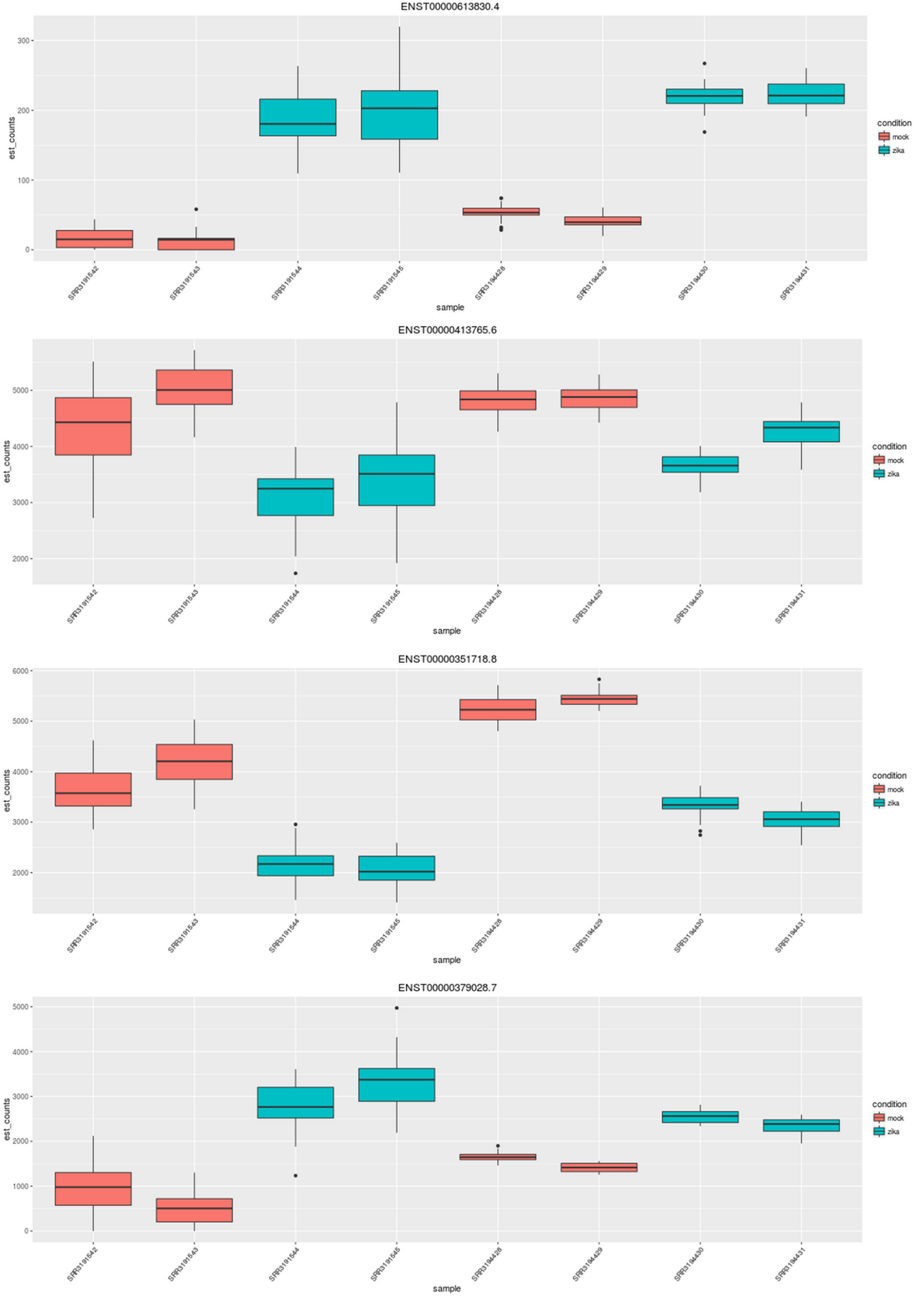
NRCAM is an example of an isoform divergent gene involved in neuron-neuron adhesion. For a specific gene, the sleuth Shiny app plots the counts corresponding to each transcript and sample. Visualized here are counts for four transcripts of NRCAM across the eight samples, colored by infection status.

**Fig 4:**
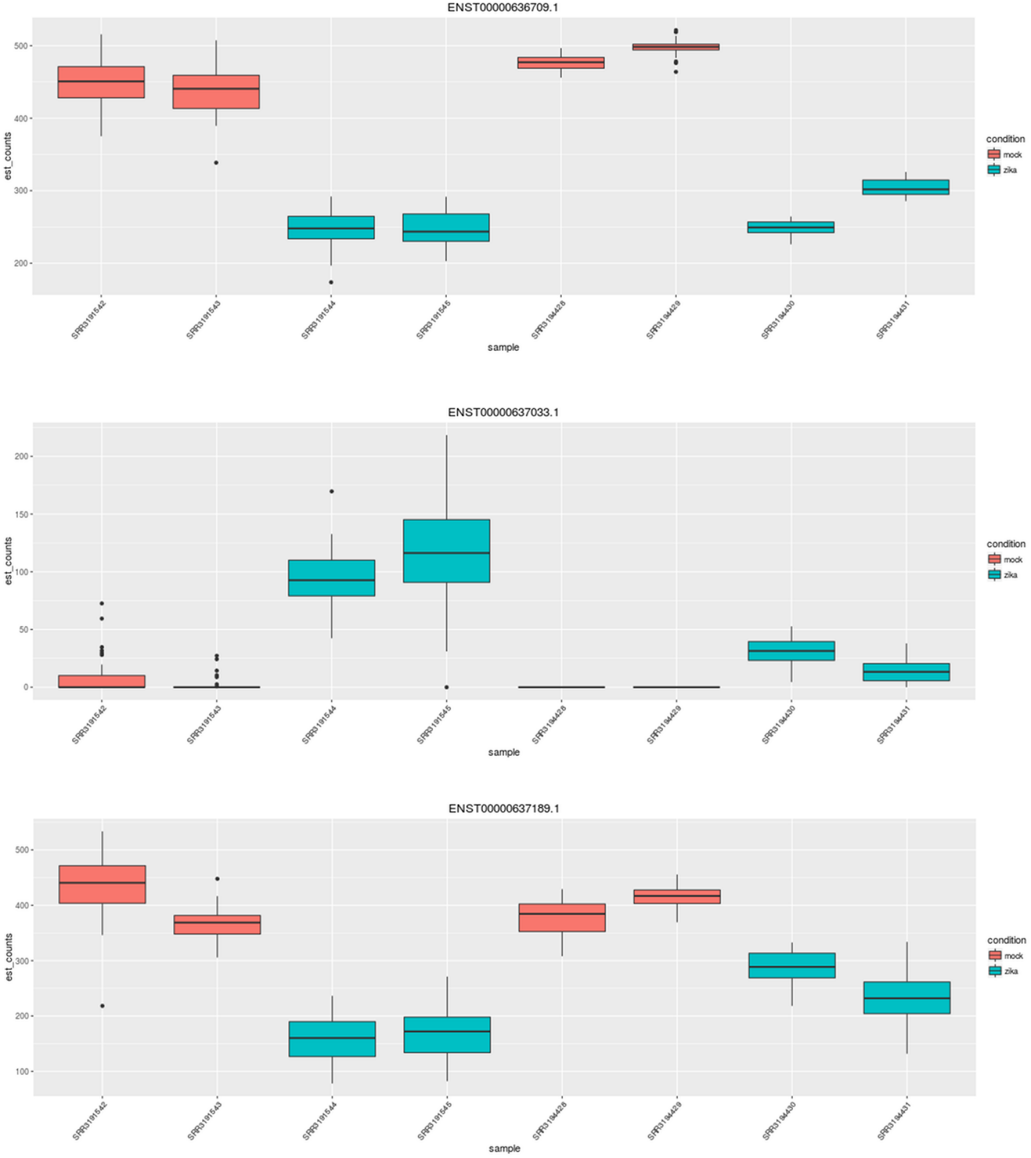
The counts of CHRNA7, another isoform diverging gene, plotted by the sleuth Shiny app. Visualized here are counts for three transcripts of CHRNA7 across eight samples, colored by infection status.

One of these 57 isoform diverging genes not picked up by Cufflink is NRCAM, neuronal cell adhesion molecule, which is putatively involved in neuron-neuron adhesion and axonal cone growth. Another is CHRNA7, cholinergic receptor nicotinic alpha 7 subunit. [8] Figs 3 and 4 shows transcript abundances in NRCAM and CHRNA7 across different samples, highlighting isoform-specific changes.

### A gene ontology (GO) analysis of sleuth-discovered genes showcase neural and head development networks

**Fig 5:**
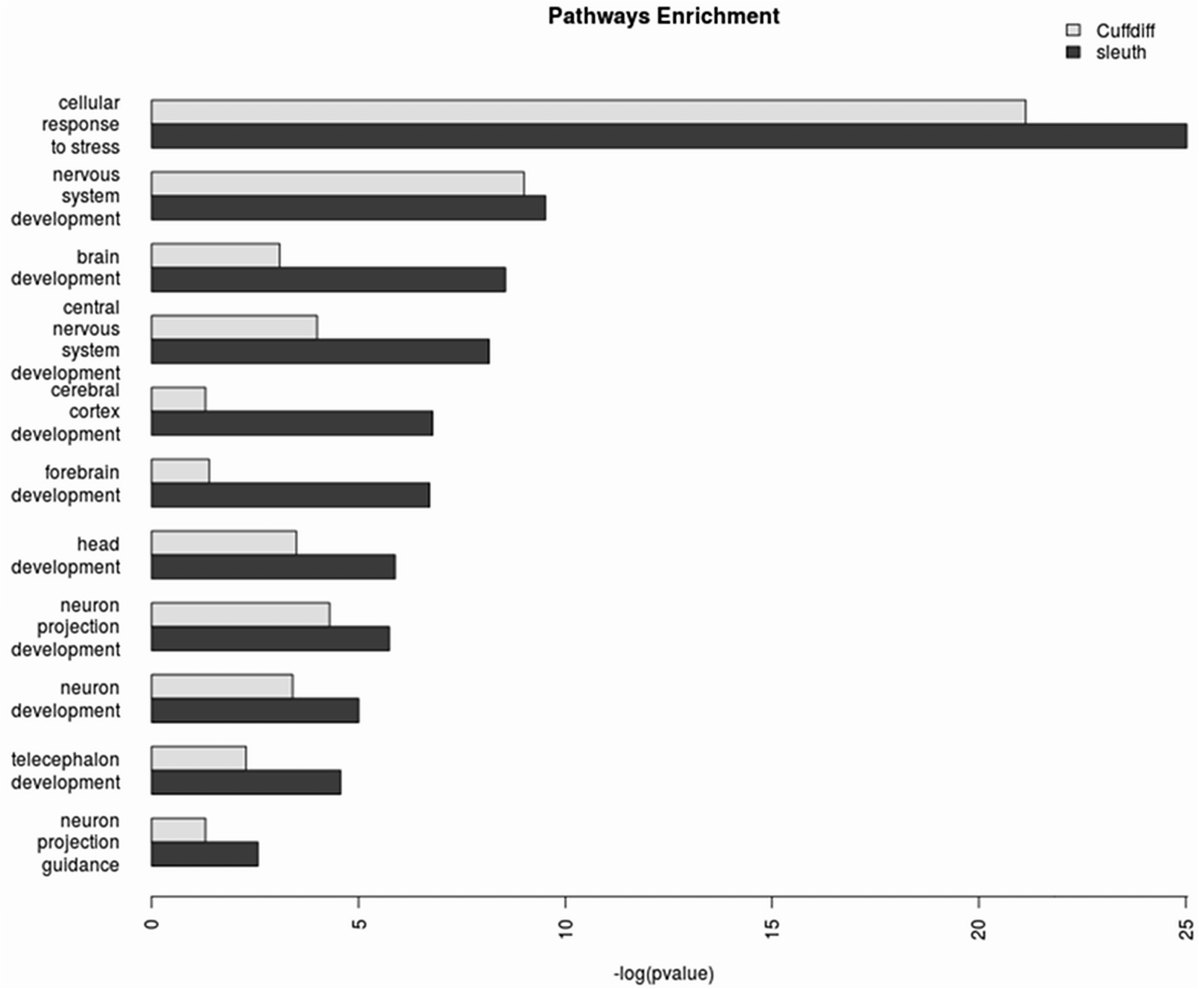
GO pathways enrichment. The enriched nodes of particular interest include neuron projection guidance (p-value = 2.7E-3 vs >0.05 with Cuffdiff), cerebral cortex development (1.6E-7 vs >0.05), neuron development (9.9E-6 vs 3.9E-4), neuron projection development (1.8E-6 vs 5.0E-5), nervous system development (3.0E-10 vs 1.0E-9), central nervous system development (6.9E-9 vs 1.0E-4), brain development (2.8E-9 vs 8.0E-4), forebrain development (1.9E-7 vs 4.1E-2), telecephalon development (2.7E-5 vs 5.2E-3), head development (1.3E-6 vs 3.2E-4), and cellular response to stress (9.4E-26 vs 7.3E-22).

We performed a side-by-side gene ontology (GO) analysis with the differential genes identified by sleuth and Cuffdiff, using ClueGO [9, 10] over the Biological Processes ontology network, using GO Term Fusion. We set the network specificity to global (GO tree interval: 1–4), using pathways with a minimum of 50 genes and kappa score of 0.5. We highlighted enriched nodes of particular interest and their enrichments in Fig 5.

Provided in the supplementary materials are the side-by-side GO analysis results tables (S4 and S5 Tables).

## Discussion

RNA-Seq can provide rapid and high resolution probing of infection response, and initial studies of Zika infection highlight isoforms, genes and pathways that may play an important role in disease etiology. However, the simplicity of RNA-Seq library prep and cDNA sequencing belies the complexity of analysis. We have shown that a careful analysis of previously published data can reveal novel targets with higher confidence, and in the process rendering a valuable dataset usable by the community of Zika researchers.

The kallisto and sleuth tools we have used in our analysis are particularly powerful when coupled with the interactive sleuth Shiny application, and our publicly available server provides access to numerous interactive plots and figures that cannot be reproduced in a static publication. This highlights the utility and importance of data sharing [11], and we hope that our analysis, aside from its usefulness for the Zika scientific community, can also serve as a blueprint for future data sharing efforts.

sleuth is a fast and accurate pipeline for analyzing RNA-Seq data that allows for testing at the isoform level. The alignment and quantification pipeline is feasible and compatible with a standard desktop computer. The interactive Sleuth application, made publically available, allows for informative data visualization, including those of library prep fragment size distributions, principle component analysis, and gene and transcript expression changes. We invite the scientific community studying Zika to utilize this toolkit.

## Supporting information

S1 Table Differentially expressed transcripts. The 4610 transcripts across 3656 genes that are found to be differentially expressed using kallisto pseudoalignment and sleuth, ordered by p-value. The columns correspond to the Ensembl transcript ID (target_id), the p-value (pval), the false discovery rate (qval), the Ensembl gene ID (ens_gene) and the gene name (ext_gene).
(CSV)

S2 Table Differentially expressed genes determined by Cuffdiff, order by p-value. A list of the differentially expressed genes (gene), their expression levels (val_1, val_2), log 2 fold change (log2.fold_change), and p-values (p_value).
(CSV)

S3 Table Isoform diverging genes. The 289 transcripts that demonstrate isoform divergence, in that at least one isoform of a gene is downregulated and at least one isoform of the same gene is upregulated. The column names are identical to those in S1 Table. There are one additional column: effect of zika infection, corresponding to the log 2 fold-change in expression levels in zika infected samples compared to mock infected samples.
(CSV)

S4 Table GO analysis results performed on Sleuth DEGs, using ClueGO on levels 1–4, showing networks with p-values <0.05. (XLS)

S5 Table GO analysis results performed on Cuffdiff DEGs, using ClueGO on levels 1–4, showing networks with p-values <0.05. (XLS)
